# Mechanistic Assessment of Norepinephrine Therapy versus Angiotensin-II in Septic Shock (MANTRA): Study Protocol for a multicenter randomized trial

**DOI:** 10.64898/2026.09.12.26362906

**Published:** 2026-09-14

**Authors:** Michael R. Filbin, Daniel E. Leisman, Ana Pachano-Bravo, Marcia B. Goldberg, Kathryn Hibbert, Olivia Nelson, Benjamin Rappaport, Simon A. Mahler, Lynnette Harris, Estelle B. Besong, Brandon Reeves, Ryan C. Maves, Andrew Petrilli, Christopher L. Schaich, D. Clark Files, Kevin Gibbs, Mark C. Chappell, Ashish K. Khanna

## Abstract

**Introduction:**

Norepinephrine remains first-line vasopressor therapy for septic shock despite its association at high doses with undesirable cardiovascular, kidney, and immune effects. Angiotensin-II (Ang-II) is a non-catecholamine vasopressor of the renin-angiotensin-aldosterone system (RAAS) now approved to increase blood pressure in vasodilatory shock. RAAS dysregulation is common in septic shock and may represent a treatable trait. However, few prospective investigations evaluate the effect of early Ang-II use in septic shock on dysfunctional RAAS biology, host-response, or clinical outcomes. Therefore, we designed a mechanistic clinical trial to test the hypothesis that early Ang-II therapy in septic shock normalizes systemic RAAS abnormalities and modulates innate immune responses.

**Methods and Analysis:**

The **M** echanistic **A** ssessment of **N** orepinephrine **T** he **R** apy vs. **A** ngiotensin-II in Septic Shock (MANTRA) trial is an investigator-initiated, prospective, multicenter, open-label, randomized mechanistic clinical trial. Adults with septic shock by Sepsis-3 criteria receiving a norepinephrine-equivalent dose (NED) of 0.10-0.50 mcg/kg/min will be randomized 1:1 to primary Ang-II (to a maximum of 40 ng/kg/min) or norepinephrine (to a maximum 35 mcg/min) treatment strategies over a 48-hour period. The primary outcome is the between-group difference in plasma renin trajectory from baseline to 24 hours, analyzed using a linear mixed-effects model with a treatment-by-time interaction term. With n=78, the trial has 90% power (at two-sided α=0.05) to detect a 25% between-group difference in 24-hour renin. Secondary clinical outcomes include longitudinal NED, hours alive and vasopressor-free at 72 hours, a 28-day composite outcome (days alive and free of vasopressors, renal replacement therapy, and mechanical ventilation), treatment failure requiring crossover or protocol discontinuation, and thrombotic and arrhythmia events through 28 days. Secondary mechanistic outcomes include serial RAAS biomarkers (prorenin, soluble prorenin receptor, angiotensinogen, ACE, ACE2, Ang-II, angiotensin-(1–7), aldosterone, and DPP3), immune measures (ex vivo monocyte function, monocyte gene expression, plasma cytokines), and organ injury biomarkers. Enrollment is planned from 5/2026-7/2030.

**Strengths and limitations of this study:**

- MANTRA is a randomized trial comparing angiotensin-II against an active standard-of-care norepinephrine strategy rather than placebo, enrolling patients earlier in septic shock than prior trials restricted to catecholamine-resistant shock.
- Serial sampling profiles RAAS peptides, innate immune function, and organ-injury biomarkers, which will generate rich mechanistic data on how vasopressor strategy shapes the host response.
- The sample size is adequate for the biomarker primary endpoints and will establish feasibility for a larger trial, but it does not provide sufficient power for hard clinical endpoints, which remain exploratory.
- MANTRA compares two open-label vasopressor strategies rather than monotherapies; clinician retain discretion over background and rescue vasopressors, steroids, and crossover, which may attenuate differences between-arms.
- The 48-hour intervention window limit inference about clinician-assessed outcomes and the durability of treatment effect.

## Full Text Availability

The license terms selected by the author(s) for this preprint version do not permit archiving in PMC. The full text is available from the preprint server.

